# Operation for the Relief of Obstruction of the Gall Duct

**Published:** 1885-02

**Authors:** 


### OPERATION FOR THE RELIEF OF OBSTRUCTION OF THE GALL DUCT.

We have received, with the compliments of the author, a well elaborated and timely article on Obstruction of the Gall Duct and its bad consequences, with remedial operation suggested, by J. McF. Gaston, M. D., of this city, which calls for something more than a passing notice.

This is a pamphlet of twenty-four pages, reprinted from Gaillard's *Journal* for October, 1884.

In this paper Dr. Gaston reviews at some length the literature of the subject, and gives his personal experience in the treatment of cases with the results of *post mortem* examinations. He concludes by suggesting a new operation, based upon his experiments with dogs for the relief of obstruction of the gall duct. This consists in making an incision into the peritoneal cavity over the most prominent part of the distended gall-bladder, when a digital or ocular examination may enable the operator to determine upon the propriety of opening the sac. Should any communication with the duodenum exist, however small it may be, dilatation of the ductus choledochus should be attempted. In the words of the author: "Should this measure be found impracticable, after evacuating the sac, whether it contains gall stone or fluid matters, a finger may be introduced and bring the surface of the alimentary canal, below the insertion of the duct, in contact with a portion of the wall of the sac, so that a needle with an elastic ligature may be passed through the tissues of both, having their adjoining surfaces united closely in the loop which constricts the inclosed walls, until they are cut through, so as to leave a communication between the two, such as has been observed in the ulcerated opening of the gall-bladder into the upper portion of the small intestine. We should avert an external fistulous discharge from the gall-bladder, by closure of its walls separately from the union of the external incision, and with the expectation that the bile shall find its way through the artificial connection into its proper channel for intermixture with the contents of the alimentary canal. There is no dcubt, on the part of physiologists, of its containing elements that are requisite for completing the digestive and assimilative processes."

The original experiments of Dr. Gaston were published in this journal, and we were present at the last operation of the series, in which the walls of the gall-bladder and duodenum were united by a single silk ligature. This was verified subsequently, as successful in effecting the communication, and we are informed that the subject of it continues in a healthy condition at present, to undergo a final exploration shortly by a third laparotomy.

Believing that this operation is destined to become an important curative agency in obstruction of the gall-duct, we deprecate the misconception of it by the *Medical Record*, of New York, for January 3d, and by the *Medical News*, of Philadelphia, for January 10th, in confounding it with the operation of Von Winiwarter for attaching the gall bladder to the colon. Gaston's operation has nothing in common with the case reported in the *Prager Medizinische Wochenschrift*, Nos. 21 and 22, 1882, in which, for all practical ends, the bile is lost to the animal economy by being deposited with the waste materials of the body; and as an operative procedure the latter has no more claim to precedence against the former than an attachment of the gall bladder to the stomach, or to the urinary bladder, or even to the abdominal wall might prefer over its union with the duodenum, which restores the bile to the natural receptacle in the alimentary canal.

Viewed from this standpoint, the suggestion is "an entirely new one," and "hence any honor and distinction which the ingenious and rational procedure merit" belongs "only to Gaston."

He proposes to establish, so far as may be possible, the normal relations; whereas, the attachment of the gall bladder to the colon diverts the bile from the channel designed for it by nature, being an abnormal disposition in like manner as its discharge by an external fistulous opening as in cholecystotomy.

The supposition of the *Medical Record* that "the external wound would either have to be kept open, or re opened after closure," in Gaston's operation, must be based upon some misapprehension in regard to the procedure, as it is taken for granted that the ligature will find its way into the intestinal canal, and that the catgut suture will ultimately be absorbed or assimilated, even if employed to close the incision of the sac after the method of Sir Spencer Wells. In any event, the external wound would not be interfered with subsequently, and is expected to unite by first intention, as clearly set forth by the author.

The statement of the *Record* that "in the present state of our knowledge cholecystotomy, as performed by Sims and Tait, still offers the best possible chances of success," leaves but a gloomy outlook for surgery in this department; and though the process of crushing gall-stones in the duct, as practiced by Tait, or of removing them, when impacted, as very recently executed by Parkes, of Chicago, meet the indications in this class of cases, no measure thus far adopted, in permanent occlusion of the gall-duct, has proved satisfactory, and Gaston's operation offers the most favorable solution of the difficulty.

### ITEMS.

Dr. Robt. Battey, of Rome, after spending several weeks in Florida, has returned to his home.

Dr. T. M. Darnall, one of the oldest physicians in Georgia, died at his home in Griffin, a few days since.

We had a pleasant call several days ago from our friend and classmate, Dr. R. H. Taylor, of Griffin, Ga.

Dr. James Knapp, a prominent physician of Louisville, died at his home, January 5th, of apoplexy.

Dr. A. W. Calhoun, of this city, has made 530 cataract operations, 96 per cent of these operations proved successful.

Diphtheria of a severe type is prevalent in New York city. The death rate from this disease is reported to be over 60 per cent.

Dr. K. P. Moore, of Macon, who was quite sick for several days in January, has so far recovered as to be able to attend his patients.

Dr. J. M. Crawford, of this city, has moved from Wheat street and is now associated with Dr. A. W. Calhoun, at 66 Marietta street.

Dr. E. C. Goodrich, of Augusta, the efficient Treasurer of the Medical Association of Georgia, has just recovered from a severe attack of diphtheria.

Dr. B. R. Dostor, of Blakely, one of the best informed physicians in Georgia, says: "I am proud of THE ATLANTA MEDICAL AND SURGICAL JOURNAL."

In the article published in our December number on "Ill-shaped Noses," etc, it was signed "A Convert," when it should have been "*A' Couvert.*"

Dr. Henry Gibons, Sr., of San Francisco, one of the most noted physicians west of the Rocky Mountains, died recently at the advanced age of seventy-six years.

The publishers announce the discontinuance of the *Archives of Medicine.* This is to be regretted, since it was one of the most valuable publications we received.

The second crematorium completed in the United States was dedicated at Lancaster, Pennsylvania, on Tuesday, November 25, at 2 p. m., when the body of a lady from Jersey City, N. J., was incinerated.

In our notice of Dr. Thomas's death, in our last issue, the typos made us say that he had gone to Washington to attend a meeting of the Ninth International Medical College. Of course it was written Congress.

*The Augusta Evening News*, one of the best and most enterprising of Georgia newspapers, says: "The Atlanta Medical and Surgical Journal is a power with the press and in the medical profession, and is ably edited."

Preservation and Protection of Cultivated Lands from Surface Washing, by David Nickols, Allatoona, Ga., has been placed on our table. From the many complimentary notices of it by farmers, we judge it to be a good book. It can be had in this city of Mr. T. F. Kent, 32 W. Alabama street.

*The Constitution.*—This enterprising daily is the pride of every Georgian, and deserves to be, since it is truly the metropolitan daily of the South. Conspicuous among its brilliant corps of editorial writers is the gifted son of the editor-in-chief, Mr. Clarke Howell, who is possibly the youngest editor in Georgia, and one of the most talented and brilliant writers on the Georgia press.

G. P. Putnam's Sons will publish early in the new year a monograph on the new anæsthetic, entitled "Cocaine and its use in Ophthalmic and general Surgery," by Dr. H. Knapp, and a treatise entitled "Acne and its Treatment," by Dr. L. B. Bulkley, a practical treatise based on the study of over 1,500 cases of diseases of the sebaceous glands.

Dr. John Charles Faget, an eminent Creole physician of New Orleans, died Sunday, December 7, 1884. His parents were *emigres* from San Domingo, and he was born in New Orleans in 1809. He was educated for medicine in Paris, and held high rank in his profession. He was the author of several standard works on yellow fever and kindred diseases, and after the epidemic of 1867 was created a Chevalier of the Legion of Honor by Napoleon III. for his services in behalf of his needy countrymen.

The University of Pennsylvania has established a department of "Physical Culture." Dr. J. William White has been elected by the trustees to preside over this new department, and his selection is a most assuring gurarantee that this very excellent departure will not be allowed to slumber. Dr. White is a believer in the equal distribution of mental and physical exercise, and he thoroughly realizes that "all work and no play will make Jack a dull boy."—*Med. and Surg. Reporter.*

Dr. Thad. A. Reamy, of Cincinnati, reported to the Academy of Medicine, December 1st, a case of fatal narcosis in an adult from one-fourth grain of morphia hypodermical y. The patient, a lady twenty-eight years old, had just undergone operation for lacerated cervix and perineum. She recovered fully and promptly from the ether. She was given the morphia by Dr. Hirons about an hour and a half after the operation, and for one hour was not impressed by the opiate. She had never taken any opium or morphia of any kind before. She died in eight hours after the administration of the morphia, despite every antidotal measure employed.

The following meteorological summary for this station for December has been kindly furnished us by S. W. Beall, Sergt. Signal Corps U. S. A.:

| | |
|---|---|
| Highest temperature, 22d | 66.5 |
| Lowest temperature, 19th | 11.0 |
| Greatest daily range of temperature, 18th | 36.2 |
| Least daily range o[illegible] temperature, 30th | 5.0 |
| Mean daily range of temperature | 15.8 |
| Mean daily dew-point | 35.9 |
| Mean daily relative humidity | 73.1 |
| Prevailing direction of wind | East |
| Total movement of wind | 8192 miles |
| Highest velocity of wind and direction, 18th | 30 miles, N. W |
| Number of foggy days | None |
| Number of clear days | 6 |
| Number of fair days | 16 |
| Number of cloudy days | 9 |
| Number of days en which rain or snow fell | 12 |
| Dates of lunar halos | 4th |
| Dates of frosts | 2d, 3d, 16th, 17th, 19th, 20th |

